# COVID-19 Pharmacotherapy: A Summary of Key Advances and Insights from the Special Issue

**DOI:** 10.3390/v15122286

**Published:** 2023-11-22

**Authors:** Georgios Schinas, Karolina Akinosoglou

**Affiliations:** 1 Department of Medicine, University of Patras, 26504 Patras, Greece; georg.schinas@gmail.com; 2 Department of Medicine and Infectious Diseases, University General Hospital of Patras, 26504 Patras, Greece

The COVID-19 pandemic has presented unprecedented challenges for healthcare systems worldwide. Beyond the development of vaccines, discovering effective pharmacotherapies to prevent and treat SARS-CoV-2 infection has been a crucial priority. This Special Issue on COVID-19 pharmacotherapy summarizes key insights into and new evidence of therapeutic options for this novel disease. Across the 17 articles published in this Special Issue, a range of drug classes are examined, including antivirals, monoclonal antibodies, immunomodulators, and supportive medications.

Several studies provide timely real-world data on novel oral antivirals like nirmatrelvir/ritonavir and molnupiravir for high-risk patients. Petrakis et al. demonstrate the effectiveness of early nirmatrelvir/ritonavir treatment in reducing hospitalizations, recovery time, and severe outcomes in unvaccinated older adults (https://www.mdpi.com/1999-4915/15/4/976, accessed on 14 November 2023). Kwok et al. find both nirmatrelvir/ritonavir and molnupiravir effective in preventing respiratory failure and mortality in unvaccinated patients with chronic respiratory diseases (https://www.mdpi.com/1999-4915/15/3/610, accessed on 14 November 2023). These findings support the use of antiviral medications as a key therapeutic strategy, in addition to vaccination, for vulnerable populations, aligning with randomized controlled trials showing efficacy in non-hospitalized patients [1,2,3]. Complementing these findings, Akinosoglou et al. provide a timely review on nirmatrelvir/ritonavir, synthesizing evidence from clinical trials and real-world data and contextualizing the value of this first-in-class oral antiviral treatment as a promising outpatient therapeutic option against COVID-19 (https://www.mdpi.com/1999-4915/14/11/2540, accessed on 14 November 2023).

Monoclonal antibodies also emerge as potential COVID-19 therapeutics, though their efficacy is threatened by viral mutations. Akinosoglou et al. review evidence showing tixagevimab/cilgavimab’s ability to prevent symptomatic infection and severe outcomes such as prophylaxis for immunocompromised patients (https://www.mdpi.com/1999-4915/15/1/118, accessed on 14 November 2023). Ren et al. discuss challenges that monoclonal antibodies face against Omicron variants and propose solutions like targeting conserved viral epitopes (https://www.mdpi.com/1999-4915/15/6/1297, accessed on 14 November 2023). Combination therapies may also improve outcomes, as Bavaro et al. find combined remdesivir and monoclonal antibodies associated with reduced COVID-19 progression in hospitalized immunocompromised patients (https://www.mdpi.com/1999-4915/15/5/1199, accessed on 14 November 2023). Finally, Ioannou et al. report that convalescent plasma may reduce rehospitalization in patients on B cell-depleting therapies (https://www.mdpi.com/1999-4915/15/3/756, accessed on 14 November 2023). Optimizing monoclonal antibodies against new variants remains an urgent priority, as their potency against emerging subvariants continues to wane [4,5,6].

Other articles investigate emerging drug classes like phenothiazines, which Liang et al. show can inhibit SARS-CoV-2 entry by targeting the spike protein (https://www.mdpi.com/1999-4915/15/8/1666, accessed on 14 November 2023). Wang et al. describe the promise of immune cell therapies to modulate inflammation and immune responses (https://www.mdpi.com/1999-4915/15/11/2148, accessed on 14 November 2023). Oliynyk et al. demonstrate the mortality benefits of medications like sildenafil (https://www.mdpi.com/1999-4915/15/5/1157, accessed on 14 November 2023) and alteplase (https://www.mdpi.com/1999-4915/15/7/1513, accessed on 14 November 2023) for COVID-19 patients with pulmonary hypertension and thromboembolism. These studies showcase active investigation into repurposed and novel medications for COVID-19, one the primary objectives of this Special Issue.

Multiple articles in this Special Issue highlight the complex interplay between COVID-19 and comorbidities like diabetes and HIV, while others synthesize evidence on key topics like pregnancy, chronic liver disease, and critical illness, thereby informing clinical management. Liontos et al. find hyperglycemia and inflammatory markers predict poor outcomes across patients with and without obesity or diabetes (https://www.mdpi.com/1999-4915/15/7/1468, accessed on 14 November 2023). Akinosoglou et al. detail COVID-19 treatment considerations unique to pregnant patients (https://www.mdpi.com/1999-4915/15/3/787, accessed on 14 November 2023), while Schinas et al. review managing CMV reactivation in critically ill patients (https://www.mdpi.com/1999-4915/15/5/1165, accessed on 14 November 2023). Furthermore, Schinas et al. examine the intersection of chronic liver disease and COVID-19 vaccination, outlining specific considerations in this patient population (https://www.mdpi.com/1999-4915/14/12/2778, accessed on 14 November 2023). Finally, Basoulis et al. delineate pharmacological management strategies and nuances when treating COVID-19 in the context of HIV (https://www.mdpi.com/1999-4915/15/2/577, accessed on 14 November 2023). These articles contextualize COVID-19 management in complex patient populations and offer insights on how customized pharmacotherapy based on comorbidity profile and disease stage may optimize COVID-19 treatment.

While vast evidence exists on certain pharmacotherapies that benefit adults with COVID-19, data on COVID-19 drug treatments in children remain far more limited overall [7,8]. Further investigation is critical to inform evidence-based treatment guidelines for these vulnerable pediatric populations. Helping to address this evidence gap, Minotti et al. conducted a retrospective study on early antiviral and monoclonal antibody treatment in high-risk children with mild COVID-19 symptoms (https://www.mdpi.com/1999-4915/15/1/192, accessed on 14 November 2023). They found that these therapies were well tolerated and associated with no cases of severe disease progression or death. This study provides valuable real-world data on the efficacy and safety of antivirals in children, as randomized controlled trials have not yet systematically included or focused on pediatric populations [9,10].

Overall, this Special Issue provides a cross-section of pharmacotherapy advances against the ever-evolving SARS-CoV-2 virus. Real-world data demonstrate the benefit of emerging antivirals for high-risk groups. However, viral resistance threatens monoclonal antibodies, requiring adapted treatment strategies. Comorbidities like diabetes markedly impact COVID-19 outcomes, emphasizing the value of personalized treatment. Repurposed medications and immune cell therapies show promise but require more investigation. Knowledge gaps remain regarding pregnancy, critical illness, and many other special populations.

Moving forward, several priorities are clear. Continuing to monitor real-world medication effectiveness as viral variants emerge is critical. Elucidating COVID-19 interactions with comorbidities is essential to optimize care, and pursuing clinical trials to expand the evidence base for novel treatments in specialized populations should remain a key goal. The work presented in this Special Issue provides a foundation to advance COVID-19 pharmacotherapy through ongoing research, enhanced treatment guidelines, and improved delivery of patient-centered care.
